# Exploring and Harnessing Haplotype Diversity to Improve Yield Stability in Crops

**DOI:** 10.3389/fpls.2017.01534

**Published:** 2017-09-05

**Authors:** Lunwen Qian, Lee T. Hickey, Andreas Stahl, Christian R. Werner, Ben Hayes, Rod J. Snowdon, Kai P. Voss-Fels

**Affiliations:** ^1^Collaborative Innovation Center of Grain and Oil Crops in South China, Hunan Agricultural University Changsha, China; ^2^Department of Plant Breeding, IFZ Research Centre for Biosystems, Land Use and Nutrition, Justus Liebig University Giessen Giessen, Germany; ^3^Queensland Alliance for Agriculture and Food Innovation, The University of Queensland, St Lucia QLD, Australia

**Keywords:** crop genomics, genomics-assisted breeding, haplotype analysis, SNP haplotype, climate change

## Abstract

In order to meet future food, feed, fiber, and bioenergy demands, global yields of all major crops need to be increased significantly. At the same time, the increasing frequency of extreme weather events such as heat and drought necessitates improvements in the environmental resilience of modern crop cultivars. Achieving sustainably increase yields implies rapid improvement of quantitative traits with a very complex genetic architecture and strong environmental interaction. Latest advances in genome analysis technologies today provide molecular information at an ultrahigh resolution, revolutionizing crop genomic research, and paving the way for advanced quantitative genetic approaches. These include highly detailed assessment of population structure and genotypic diversity, facilitating the identification of selective sweeps and signatures of directional selection, dissection of genetic variants that underlie important agronomic traits, and genomic selection (GS) strategies that not only consider major-effect genes. Single-nucleotide polymorphism (SNP) markers today represent the genotyping system of choice for crop genetic studies because they occur abundantly in plant genomes and are easy to detect. SNPs are typically biallelic, however, hence their information content compared to multiallelic markers is low, limiting the resolution at which SNP–trait relationships can be delineated. An efficient way to overcome this limitation is to construct haplotypes based on linkage disequilibrium, one of the most important features influencing genetic analyses of crop genomes. Here, we give an overview of the latest advances in genomics-based haplotype analyses in crops, highlighting their importance in the context of polyploidy and genome evolution, linkage drag, and co-selection. We provide examples of how haplotype analyses can complement well-established quantitative genetics frameworks, such as quantitative trait analysis and GS, ultimately providing an effective tool to equip modern crops with environment-tailored characteristics.

## Introduction

Unstable environments and increasing climatic fluctuations like extreme heat and drought events have a severe impact on global crop production (Lesk et al., 2016). In order to meet the food demand of a rapidly growing world population, yields of important commodity crops need to be increased by almost 40% by the middle of this century (Tester and Langridge, 2010). Breeding of cultivars that consistently achieve high yields even in inconsistent environments is a highly challenging task for the global plant breeding community. The enormous recent advances in DNA sequencing and genotyping technologies may help to overcome this challenge. Low-cost genotyping tools that can capture sequence variation at ultra-high resolution are now available for all agronomically important plant species (Huang and Han, 2014) and commercial genotyping platforms that can generate thousands or millions of data points per genotyping experiment are now in standard use in genomic research (Bassi et al., 2016). These powerful tools provide an effective means for crop genetic research studies (Ganal et al., 2012), for example enabling the assessment of population structure and genotypic diversity on a genome-wide, subgenome-wide, or chromosome-wide scale, facilitating identification of selective sweeps and signatures of directional selection, or providing a basis for genomic selection (GS) or prediction of hybrid performance.

Targeting genetic variants associated with agronomic traits and identifying important underlying candidate genes have become a key area in crop genetic research (Voss-Fels and Snowdon, 2016). Because the majority of high value traits are quantitatively inherited, in recent decades considerable focus has been placed on mapping and characterization of quantitative trait loci (QTL). Quantitative trait loci mapping remains a powerful method to identify genes with major effects, however, for highly complex traits its usefulness is limited because bi-parental mapping populations lack genetic diversity [possessing only two alleles at any given single-nucleotide polymorphism (SNP) locus] and provide only a low mapping resolution due to a lack of recombination events [Brachi et al., 2011; for further methodical details of QTL mapping see Collard et al. (2005)]. Along with the advances in genotyping technologies, huge progress has been achieved in the development of statistical solutions to handle vast amounts of genomic data. Although high-density data provide little additional information in a bi-parental context, genome-wide association studies (GWASs), also known as linkage disequilibrium (LD) mapping using high-density molecular marker information have become a widely used and powerful tool for the genetic dissection of complex traits at an extremely high resolution [see Zhu et al. (2008) for status and prospects of GWAS in plants]. Simultaneously, high-density marker data in genetically diverse plant populations can also provide valuable information about structural diversity of crop genomes.

Typically, high-throughput SNP marker arrays or SNPs detected by reduced-representation DNA sequencing are the genotyping methods of choice for crop genomic investigations. However, in the context of GWAS, SNPs have the major limitation that they normally only provide biallelic information at any individual locus. Further, assuming the rare allele model (Gibson, 2012), a significant fraction of the genetic variance for a given quantitative trait is explained by rare alleles (Maher, 2008) that are often not adequately represented on commercial genotyping arrays and are therefore difficult to detect (Wray et al., 2013; Voss-Fels and Snowdon, 2016). On the other hand, as a consequence of natural and artificial directional selection, most important crop species show abundant levels of LD (Gupta et al., 2005). Thus, QTL identified by GWAS is unlikely to represent true causal molecular variants, but rather loci that are in LD with a gene or a regulatory element that affects the trait of interest (Mackay et al., 2009; Korte and Farlow, 2013). An effective approach to overcome the biallelic limitations of SNPs and to increase the allelic resolution of candidate genomic regions is to employ haplotypes, the specific combination of jointly inherited nucleotides or markers from polymorphic sites in the same chromosome segment (Stephens et al., 2001; Lu et al., 2010).

Here, we review the latest advances in haplotype analyses in crops, highlighting their potential for the improvement of yield stability in the face of increasing abiotic constraints to crop production. We discuss technical aspects of haplotype description using high-throughput genotyping platforms and how these can be useful for genomics-based crop improvement. We also discuss the importance of haplotypes in the context of polyploidy, co-selection, and linkage drag, and give future perspectives on how they can be incorporated in established and emerging quantitative genetics frameworks, such as quantitative trait analysis and GS.

## Definition and Formation of Haplotypes

Bernardo (2010) describes a haplotype as “two or more SNP alleles that tend to be inherited as a unit.” However, the construction of haplotypes based on empirical marker data is not trivial (Korte and Farlow, 2013). Wall and Pritchard (2003) describe key methods to define haplotypes by (i) taking haplotype diversity of a given chromosomal segment as a basis for haplotype assignment or (ii) by using approaches that assign haplotypes based on pairwise LD between markers showing little or no evidence of historical recombination and a joint inheritance in the same a chromosomal block, typically measured as *r*^2^ (Pritchard and Przeworski, 2001). Linkage disequilibrium-based approaches are advantageous because (i) they focus directly on the detection of historical recombination in a given population, via identification of haplotype blocks, (ii) they are easily applicable in diploid data in which the haplotype phase is unknown, and (iii) LD coefficients are easy to visualize. There are numerous factors affecting LD, such as the propagation type and the rate of inbreeding in a given species, the size of the analyzed population, the extent of population stratification and subdivision, genetic drift, recombination frequency, mutation rate, or the strength and type of selection on a given chromosomal fragment (Gupta et al., 2005).

Historically, directional selection for genes or alleles conferring favorable characteristics (Lande and Arnold, 1983) has played a major role in the formation of signatures of selection in all globally important crop species, and numerous examples have been reported for example in rice (*Oryza sativa* ssp. *japonica*, ssp. *indica* and their progenitor *O. rufipogon*; Flowers et al., 2012), maize (*Zea mays* ssp. *mexicana*; Hufford et al., 2013), wheat (*Triticum aestivum* L.; Voss-Fels et al., 2015), sorghum (*Sorghum bicolor* L. ssp. *bicolor*; Mace et al., 2013), cassava (*Manihot esculenta* L.; Wang et al., 2014), or rapeseed (*Brassica napus* L.; Qian et al., 2014). Signatures of selection, also referred to as selective sweeps or conserved haplotype blocks, typically contain numerous genes and it is likely that their expression is jointly controlled by multiple regulatory alleles, resulting in trait correlations that are more likely to be caused by linked genes rather than pleiotropy (Qian et al., 2016a). Targeting these selection hotspots, unraveling their underlying effects on relevant traits, and increasing recombination by genomics-driven crossing strategies could greatly improve our understanding of quantitative trait complexes and improve modern varieties with tailored agronomical characteristics and better adaptation to extreme climates.

## GWAS-Based Haplotype Analysis

**FIGURE 1:**
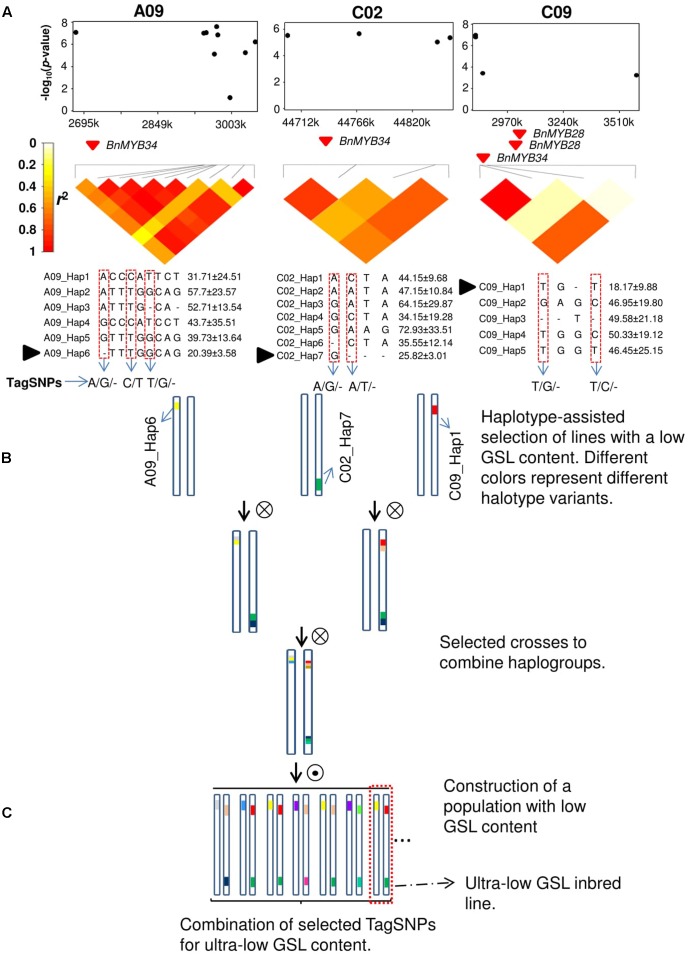
Haplotype-based marker-assisted selection; ultra-low glucosinolate (GSL) content in rapeseed as an example. **(A)** GWAS results show three different haplotypes on chromosomes A09, C02, and C09 which are significantly associated with GSL content. TagSNPs were identified in three haplotype regions. Black solid triangle represents favorable haplotype alleles contributing to the lowest GSL content. **(B)** Selected accessions based on these three favorable haplotype alleles in three different haplotype regions and crossing schemes to combine these alleles in a population. **(C)** TagSNPs combination with phenotypic screening of ultra-low GSL inbred lines in a population with low GSL content. The dotted box represents an ideal ultra-low GSL inbred line.

Since the advent of highly efficient genotyping technologies, GWAS has become the method of choice for the dissection of complex traits in humans, animals, and plants. Numerous examples have demonstrated the power of GWAS for dissection of agronomically relevant quantitative traits in all major crops (Huang and Han, 2014). The most frequently used markers for GWAS are SNPs and commercial genotyping platforms can rapidly provide robust and cheap genome-wide marker data for all important crop species. Single-nucleotide polymorphism markers are typically biallelic and show a much lower mutational rate and lower per-marker information content than other previously used, single-plex marker systems such as simple sequence repeat (SSR) markers (Würschum et al., 2013). Further, it is well documented that significant SNPs in GWAS experiments are unlikely to represent causal molecular variants due to inherent ascertainment biases of SNP arrays and the fact that extreme phenotypes are frequently caused by rare alleles (Wray et al., 2013). Due to genetic heterogeneity, non-causative markers in LD with a true molecular variant can show a more significant statistical marker–trait association than the causal variant itself (Platt et al., 2010; Korte and Farlow, 2013). Nevertheless, such false-positive synthetic associations can still be a valuable predictor of the phenotype if LD with the causal variant is strong. However, in most cases the resolution at which complex relationships between quantitative phenotypes can be depicted with biallelic SNPs is limited. An efficient way to overcome this problem and to increase allelic variation along sites in the genome that are significantly associated with phenotypes is by analysis of haplotype blocks in target regions exhibiting trait associations, as shown in **Figure 1**. Here, candidate regions are identified by GWAS or QTL mapping and local LD surrounding the identified markers is analyzed (**Figure 1A**). Based on LD criteria, marker-based haplotypes consisting of multiple markers are built and their allelic variation is compared to phenotypic measures for the traits of interest. Subsequently, test lines carrying favorable alleles can be screened and selected for crossings using molecular markers in order to accumulate haplotype variants of interest in breeding lines (**Figures 1A,B**).

Stephens et al. (2001) showed that haplotype blocks combining two or more SNPs in strong LD show a highly increased heterozygosity due to their inherent multiallelic nature and are thus much more informative than single biallelic SNPs. That study revealed a higher number of haplotype variants than SNP markers, indicating recombination and recurrent mutation events within and among the genes in the haplotype. In recent years, haplotype analyses on the basis of genome-wide association scans using SNP markers have successfully been applied in a wide range of crop species to identify important candidate genes for various traits. In maize (*Z. mays* L.), for example, Yang et al. (2013) confirmed via GWAS that a CO, CO-LIKE and TOC1 (CCT) domain-containing protein is a main factor for photoperiod response. After locating the candidate gene in a highly conserved selective sweep with pronounced levels of LD, they were able to identify different haplotype variants in the promoter region with highly specific corresponding phenotypes. They further showed that a transposable element in the gene promoter region dramatically reduced flowering time, a phenotype which likely accelerated the worldwide dispersal of maize production into regions characterized by long-day environments. In rice (*O. sativa* L.), Si et al. (2016) mapped a highly significant SNP in a gene for grain size via GWAS. By resequencing the candidate region they subsequently identified two major haplotypes that corresponded to small or long grain phenotypes, respectively. Another recent rice genetics study used ultra-dense SNP marker information from whole-genome resequencing data of 176 rice varieties to identify novel genes with major effects on agronomically important traits (Yano et al., 2016). Deploying information from almost 430,000 polymorphic SNPs and 68,000 insertion–deletion markers, they designed a four-step experimental process for rapid gene identification including (i) significance testing for marker signals using GWAS, (ii) definition of candidate haplotype regions with significant markers on the basis of LD, (iii) extraction of candidate genes based on annotation information, and (iv) validation of functional haplotype alleles through introgressions. In wheat (*T. aestivum* L.), Jiang et al. (2015) analyzed molecular variants of three main cell wall invertase (CWI) genes that were found to have a significant effect on grain yield. By performing haplotype analyses based on SNP and insertion–deletion markers in the gene coding sequences they demonstrated that the haplotype variants with positive effects on grain yield were most frequent in modern cultivars and were strongly positively selected in six major global wheat growing regions. Using haplotype network analyses, Jiang et al. (2015) demonstrated that strong allelic selection in the course of breeding, domestication, and polyploidization has narrowed the genetic diversity of three major wheat CWI genes.

## Multi-Trait Haplotypes and Selective Sweeps

Crop breeders have been tremendously successful in combining beneficial loci within germplasm, resulting in an enhancement of yield throughout the history of modern agriculture (Mickelbart et al., 2015). Examples demonstrating the effectiveness of genetic-driven yield increases have been given in wheat (*T. aestivum* L.) (Austin et al., 1980; Cormier et al., 2013; Laidig et al., 2017a); maize (*Z. mays* L.) (Tollenaar, 1989), rye (*Secale cereale*) (Laidig et al., 2017b), rice (*O. sativa* L.) (Zhu et al., 2016), triticale (×*Triticosecale*) (Losert et al., 2017), and oilseed rape (*B. napus* L.) (Kessel et al., 2012; Stahl et al., 2017). Paradoxically, however, the tremendous success of selective breeding has also caused a drastic reduction of genetic diversity in many elite crop germplasm pools, limiting the potential for future genetic gain. It is well documented that strong directional selection, either naturally through local adaptation or artificially through breeding, has resulted in pronounced LD patterns across crop genomes. There are numerous examples of selective sweeps and signatures of selection in important crop species, visible as highly conserved haplotype blocks with low levels of recombination (Voss-Fels and Snowdon, 2016).

This is especially critical for future breeding success, as a reduction in genetic diversity ultimately leads to a decrease of long-term breeding progress. To secure continuous breeding success, constant replenishment of genetic diversity is essential (Govindaraj et al., 2015). On the other hand, maintaining a sufficient effective population sizes while minimizing negative effects through linkage drag is challenging (Cowling, 2007; Cowling and Léon, 2013; Griggs et al., 2014). In general, crop gene banks represent a rich diversity of haplotype variants, typically much broader than in commercial breeding programs (Mackay et al., 2016; Riaz et al., 2017). A classic example of the conflict between breeding success and gene pool diversity is seen in oilseed rape/canola (*B. napus* L.), today the second most important oilseed crop in the world. The recent allopolyploid *B. napus* arose only recently from a small number of hybridization events between *B. rapa* and *B. oleracea*, and wild forms do not exist. Today’s elite breeding pool was narrowed even further by introduction of the essential seed quality traits “low erucic acid” and “glucosinolate content” from just two trait donors, causing another extreme genetic bottleneck (Hasan et al., 2006; Qian et al., 2014). This resulted in very large, extremely conserved chromosomal blocks in all major ecogeographic gene pools of canola and oilseed rape (Qian et al., 2014), accompanied by low effective population sizes in elite germplasm collections (e.g., Cowling, 2007) and hidden effects of linkage drag. For example, Qian et al. (2016b) discovered a conserved haplotype block in which a gene associated with reduced glucosinolate content caused linkage drag among variants of functionally independent chlorophyll-related genes. This resulted in phenotypic correlations between seed glucosinolate and leaf chlorophyll content that appeared to have negative effects on photosynthesis and oil accumulation in some stress environments. In other species, several mapping studies have shown pleiotropic actions of identified QTL on multiple crop characteristics, for example in rice (*O. sativa* L.) (Bai et al., 2010) and maize (*Z. mays* L.) (Bomblies and Doebley, 2006). More precise identification of gene variants in haplotype blocks based on growing quantities of pangenomic sequence data will provide a basis for more precise selection of environmentally resilient cultivars, for example using high-density molecular markers. The higher allelic resolution of identified candidate regions will sustain a more accurate delineation of complex marker–trait correlations and help to significantly improve resilience of future cultivars to extreme weather conditions, such as heat and drought (Lesk et al., 2016). Whereas adaptation to abiotic constrains was previously mainly achieved by combination of anonymous loci and relatively inaccurate phenotypic selection, the identification of genetic determinants will become increasingly important in future postgenomic approaches aiming to speed up breeding progress by targeted selection (Mickelbart et al., 2015).

## Homeologous Genome Regions and Haplotype Interactions

Many globally important crops are recent or ancestral allopolyploids, yet the direct effect of polyploidy on evolutionary success in recent species is largely unknown. In the formation of many important polyploids, such as wheat (*T. aestivum* L), cotton (*Gossypium arboreum* L.), tobacco (*Nicotiana tabacum* L.), or oat (*Avena sativa* L.), hybridizations between related species merged genomic information from two or more divergent relatives into a single nucleus. Immediate or subsequent genome restructuring and gene loss (Chalhoub et al., 2014; Samans et al., 2017) resulted in divergence from expected genomic additivity with strong consequences for crop evolution and adaptation (Soltis et al., 2009; Mason and Snowdon, 2016). There is growing evidence that duplications and/or deletions of chromosome segments contributed to genetic variation which modulates expression of homoeologous genes. This resulted in phenotypic variation which may sustain adaptation and domestication of polyploid species (Chen, 2007). For example in allotetraploid cotton (*G. arboreum* L.), unequal distribution and expression bias of homoeologous genes as a consequence of asymmetric subgenome evolution were found to be associated to fiber development and a wider environmental adaptation (Zhang T. et al., 2015). In *B. juncea* it was proposed that expression dominance of homoeologous genes facilitated directional selection for genes associated with seed glucosinolate content and lipid metabolism in modern varieties used for food and oil production (Yang et al., 2016). Directional selection for specific homoeologous genetic variants increases the frequency of favorable alleles in a population and affects their standing variation, resulting in the formation of conserved haplotypes with strong surrounding LD. Comparing and dissecting these homoeologous chromosome blocks, while taking into account evolutionary and domestication processes, can improve our understanding of complex genetic mechanisms underlying quantitative traits. For example, a population-scale resequencing approach of a diverse collection of hexaploid wheat (*T. aestivum* L.) lines found that duplicated homoeologous genes are under purifying selection, implying that a fitness benefit could be obtained by a mutation at any one of the homoeologs and that directional selection likely acted on single advantageous mutations in homeologous chromosome regions (Jordan et al., 2015). In rapeseed (*B. napus* L.), a GWAS-based haplotype analysis found that duplicated homoeologous haplotype regions in the two subgenomes additively affect leaf chlorophyll content and specific variants have been co-selected in the same direction (Qian et al., 2016a). These findings indicate that haplotype analyses can shed new light on phenotypic effects of structural variations among homoeologous regions. Use of LD-based haplotypes to more accurately distinguish between homologous loci can therefore improve our understanding of the consequences of artificial selection on quantitative trait complexes, enhancing the potential for genomics-driven improvements of future cultivars.

Two important genetic features of the variance of complex traits in crops are additive and epistatic effects (Lamkey et al., 1995; Goldringer et al., 1997; Ma et al., 2007). While QTL often additively explain a fraction of the phenotypic variation, different studies have reported epistatic QTL interactions for various important crops and traits, including wheat (*T. aestivum* L.) (Zhang et al., 2008; Li et al., 2014), rice (*O. sativa* L.) (Wang et al., 2012; Chen et al., 2014), maize (*Z. mays* L.) (Ma et al., 2007; Bocianowski, 2013), and rapeseed (*B. napus* L.) (Basunanda et al., 2010; Shi et al., 2011). Since the advent of modern high-throughput genotyping technologies which significantly increased the genomic resolution and the establishment of GWAS techniques, significant phenotypic effects of epistatic SNP–SNP interactions on various complex traits have been reported in humans (Lindstrom et al., 2011; Ma et al., 2015), animals (Bolormaa et al., 2013; Ali et al., 2015), and crops (Snowdon et al., 2015; Zhang J. et al., 2015). SNP–SNP interactions normally cannot represent intergenic interactions at the single gene level and the exact components for genetic variation of quantitative traits affected by multiple genes in shared biological pathways are elusive. However, selection-driven LD in haplotype blocks and the simultaneous consideration of various multi-locus marker alleles allows a more precise and powerful detection of epistatic interactions between unlinked loci at different locations in the genome.

**FIGURE 2:**
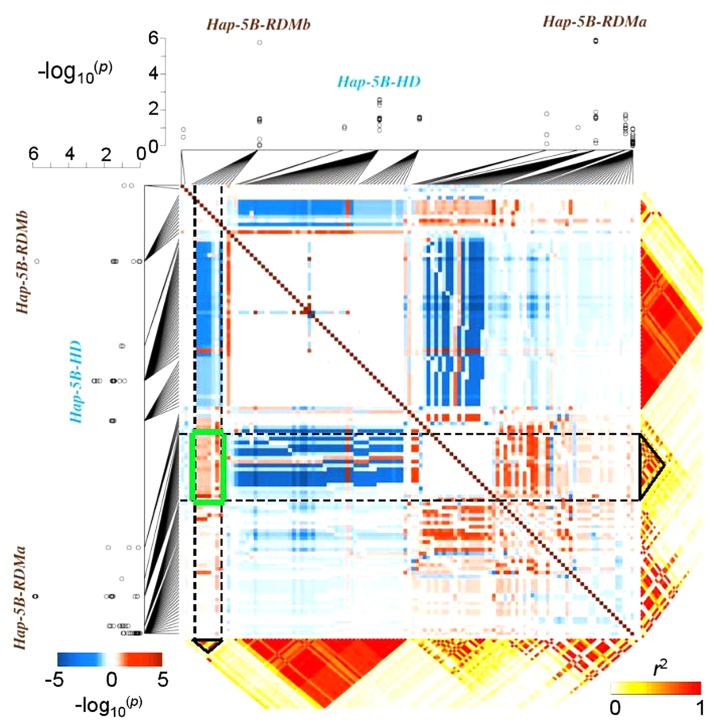
Epistatic interactions among co-selected haplotypes: an example from bread wheat. Genome-wide association mapping results showing the haplotypes *Hap-5B-RDMa* and *Hap-5B-RDMb* which are significantly associated with root dry mass in 215 wheat inbred lines. The squared heat map (blue–red) shows the –log_10_-(*p*-values) of the SNP × SNP interaction effects of the SNPs located in haplotype blocks; a negative sign indicates a negative epistatic interaction. SNP × SNP epistatic interactions were confirmed by the epistatic interaction test implemented in the whole-genome association analysis toolset PLINK (v1.07, http://pngu.mgh.harvard.edu/purcell/plink/). The green box highlights a region with strong positive SNP × SNP epistatic between these two haplotypes. Modified from Voss-Fels et al. (2017) and reprinted with permission from John Wiley and Sons ©2017.

Different molecular genetic studies have attempted to model the effect of epistasis on quantitative traits. For example, interactions among molecular variants (often rare alleles) play a major role in determining the susceptibility of humans to a particular disease. Li et al. (2010) proposed a model to map haplotype × haplotype interactions responsible for human diseases. Similar approaches have been adapted to crops, demonstrating that interactions between haplotypes can significantly influence agronomic traits. Prominent examples are the vernalization response in barley (*Hordeum vulgare* L.) (Cockram et al., 2007), yield in wheat (*T. aestivum* L.) (Qin et al., 2014), or chlorophyll content in rapeseed (*B. napus* L.) (Qian et al., 2016a). A recent study showed that haplotype-based approaches are also useful to disclose relationships between crop characteristics that appear to be unrelated, for example inflorescence development and root growth in wheat. By mapping two highly significant haplotypes for root biomass in close proximity to a major locus known to affect spike development, Voss-Fels et al. (2017) uncovered selection-driven linkage drag and strong LD causing inadvertent co-selection of haplotype variants associated with low root biomass in European elite wheat germplasm (**Figure 2**). Using a linear model to estimate pairwise SNP–SNP interaction effects on root biomass, strong positive epistatic interactions were detected among root-associated haplotypes and negative effects were detected for interactions between LD blocks associated to spike and root traits. High-resolution SNP and LD data are an ideal basis for breeders to disrupt linkage in repulsion among valuable traits, particularly (as for root traits) where phenotypic selection is challenging.

## Haplotype-Based Improvement of Molecular Breeding Approaches

**FIGURE 3:**
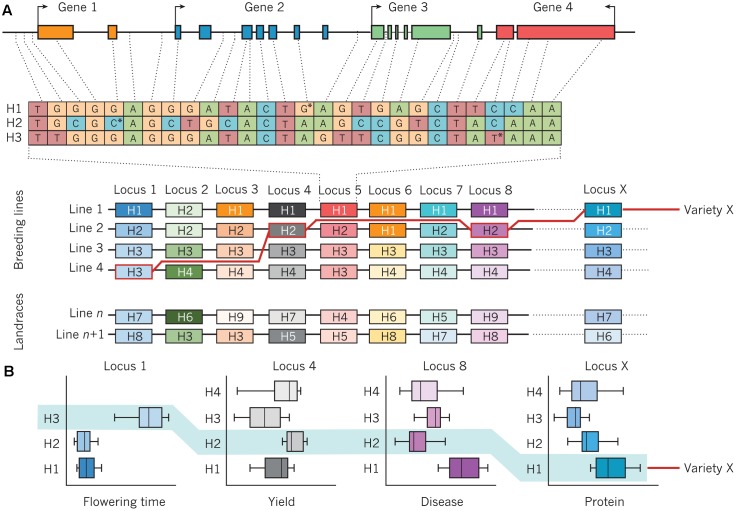
Assembly of haplotypes in a crop-breeding program. **(A)** An example of a genomic region that consists of four genes and contains genetic variation that defines three haplotypes (H1, H2, and H3) at a particular locus (locus 5) on a chromosome. The position of the SNP that defines each haplotype is marked by an asterisk. An array of haplotypes (H1–H4) from the same chromosome, with the variants of four breeding lines (line 1, line 2, line 3, and line 4) aligned underneath each locus, is also shown. Line *n* and line *n*+1 are landraces (domesticated lines) that can introduce new haplotypes (H5–H9) and genetic diversity. The genomic structure, diversity, and functions of haplotypes are established by the re-sequencing of lines and the analysis of quantitative trait loci. The red line traces the assembly of a new line (variety X) from component haplotypes, using markers that are specific for the haplotypes in each line, that have been chosen on the basis of desired combinations of phenotypes that are expressed by each haplotype. **(B)** The performance of various haplotypes in lines 1–4 is determined in different environments, often under field conditions and over several years, using specific assays. Examples are shown for the variation in performance of four common plant traits that are influenced by genetic variation at locus 1 (time to flower), locus 4 (yield), locus 8 (resistance to disease), and locus X (protein content), with the combined performance of variety X highlighted in light blue. Figure from Bevan et al. (2017) and reprinted with permission from Nature Publishing Group ©2017.

Improving the resilience of modern crop cultivars in times of increasing climatic fluctuations is a major challenge for breeders (Massawe et al., 2016). Although marker-assisted selection (MAS) could effectively be deployed for traits with a mono- or oligogenic inheritance, selection of highly quantitative traits with a low heritability due to strong environmental influence remains challenging. In this context, MAS strategies have largely failed due to statistical overestimation of QTL-linked markers or complex genetic architectures for most important traits (Bernardo, 2008). To overcome this problem, the concept of GS with densely spaced genome-wide markers is presently being adopted for many crop breeding programs. Genomic selection methods, initially conceived for animal breeding (Meuwissen et al., 2001), apply the concept that the breeding value of an individual which has not been phenotyped can be estimated purely on the basis of its genome-wide marker profile. Details about methodical concepts of GS and examples of the application of GS in crop breeding are given by Jannink et al. (2010). Given the strong genome structure present in the breeding pools of most crops, the deployment of haplotypes could be a powerful complementary tool to improve accuracy and efficiency of both MAS and GS. Varshney et al. (2005) recognized the high potential of haplotypes for whole-genome selection, based on comprehensive haplotype maps to identify and utilize genome regions are associated with trait of interests in populations with pronounced LD structures. Bevan et al. (2017) refer to retrospective and prospective approaches to implement haplotypes in genomics-assisted breeding (**Figure 3**). In the retrospective approach, favorable haplotypes that have been selected by breeders for a given crop species are identified by first resequencing the genomes of key genotypes with high importance and related, derived breeding lines which have been evaluated across multiple environments and years, as shown in **Figure 3A**. This provides retrospective information about selection decisions made by breeders, identifying genomic haplotypes associated with previous breeding success. These can be used to identify causal gene candidates and underlying networks, as well as desired and deleterious genetic variants that cause specific phenotypes. Subsequently, as shown in **Figure 3B**, molecular markers that define favorable haplotype blocks can be jointly selected to create novel combinations of haplotypes with well-defined trait effects. Haplotype-related markers can also be used to identify lines with novel recombination in chromosomal blocks of interest in order to separate favorable and unfavorable genetic variation. The definition of genome-wide haplotypes by specific functional and genetic relationships differs from classical GS, where genome-wide markers represent anonymous components that are statistically related to phenotypes in a subset of the total breeding population. Instead, Bevan et al. (2017) suggest prospective approaches for haplotype utilization in genomics-based crop improvement through resequencing of large ancestral and wild relative populations of a given crop species, in order to identify haplotypes with a broader range of genetic variation than is present in elite breeding pools. The aim is to define new haplotypes with fewer deleterious genetic components, and subsequently use molecular markers to define haplotype blocks which can be incorporated into breeding programs. A recent study in rice (*O. sativa* L.) showed that the prediction accuracies for different traits using classical GS models like ridge-regression best linear unbiased prediction (RR-BLUP) could significantly be improved by a model extension based on upstream GWAS analyses (Spindel et al., 2015, 2016). Using a collection of 369 elite rice breeding lines, Spindel et al. (2016) mapped significant SNP markers for various traits (e.g., flowering time and plant height, recorded in multiple environments) and fit these markers as fixed effects in a RR-BLUP model (referred to as a GS + *de novo* GWAS model). They showed that extended GS + *de novo* GWAS models outperformed six other prediction models for various traits. Interestingly, the prediction accuracies of extended GS models outperformed classical models using phenotype data from dry seasons, implying that this approach is particularly suitable for the improvement of drought-resistance in future crop cultivars. Because of their increased information content compared to biallelic SNP markers, fitting haplotypes with statistically significant trait associations to phenotypes as fixed effects in GS models could further improve prediction accuracies. The use of haplotype-assisted GS should more accurately depict the complex relationships between genotypic information and phenotypes than single SNPs alone are able to do; hence, this approach could ultimately help to further increase selection gain per unit of time. Also in horticultural crops, the deployment of haplotype-based approaches holds a great potential for a genetic improvement of future varieties. In cucumber (*Holothuria edulis* L.) for example, Soliman et al. (2016) used PCR-based markers to study sequence diversity in a mitochondrial cytochrome oxidase subunit I (COI), 16S ribosomal RNA, and a nuclear histone. Applying marker-based haplotype analyses they were able to gain insights into population structure, providing valuable information for the management of sea cucumber populations in Okinawa. In grapevine (*Vitis vinifera* L.), Fernandez et al. (2014) analyzed haplotype variation in a gene homologous to *TERMINAL FLOWER 1* from *Arabidopsis thaliana* and found a relationship between haplotype diversity and phenotypic variation for reproductive and inflorescence traits. Gavrilenko et al. (2013) used SSR markers developed from plastid genome sequences to investigate the genetic relationships between cultivated and wild potato (*Solanum tuberosum* Andigenum and *S. tuberosum* ssp. *andigenum*) species. By analyzing haplotype diversity they could gain further insights into the origin of cultivated potatoes. Combining advanced statistical genetics approaches with ultra-fast crop propagation techniques that allow multiple plant generations per year under greenhouse conditions (Riaz et al., 2016) could help to rapidly accelerate environmental resilience of modern crop cultivars to secure future food supply.

## Author Contributions

LQ, KV-F, and RS conceived the review; LQ, KV-F, AS, and RS conducted the literature survey and drafted the article; LQ and KV-F wrote the manuscript; and LH, CW, and BH contributed additional concepts and carried out critical revision of the manuscript.

## Conflict of Interest Statement

The authors declare that the research was conducted in the absence of any commercial or financial relationships that could be construed as a potential conflict of interest.
